# Molecular epidemiology and pathogenomics of extended-spectrum beta-lactamase producing- *Escherichia coli* and - *Klebsiella pneumoniae* isolates from bulk tank milk in Tennessee, USA

**DOI:** 10.3389/fmicb.2023.1283165

**Published:** 2023-11-06

**Authors:** Benti D. Gelalcha, Ruwaa I. Mohammed, Aga E. Gelgie, Oudessa Kerro Dego

**Affiliations:** ^1^Department of Animal Science, The University of Tennessee, Knoxville, TN, United States; ^2^Department of Genome Science and Technology, The University of Tennessee, Knoxville, TN, United States

**Keywords:** ESBL, *Escherichia coli*, *Klebsiella pneumoniae*, sequence types, virulence gene, high-risk clones, bulk tank milk

## Abstract

**Introduction:**

The rise in extended-spectrum beta-lactamase (ESBL)-producing *Enterobacteriaceae* in dairy cattle farms poses a risk to human health as they can spread to humans through the food chain, including raw milk. This study was designed to determine the status, antimicrobial resistance, and pathogenic potential of ESBL-producing -*E. coli* and -*Klebsiella* spp. isolates from bulk tank milk (BTM).

**Methods:**

Thirty-three BTM samples were collected from 17 dairy farms and screened for ESBL-*E. coli* and -*Klebsiella* spp. on CHROMagar ESBL plates. All isolates were confirmed by matrix-assisted laser desorption ionization time-of-flight mass spectrometry (MALDI-TOF MS) and subjected to antimicrobial susceptibility testing and whole genome sequencing (WGS).

**Results:**

Ten presumptive ESBL-producing bacteria, eight *E. coli*, and two *K. pneumoniae* were isolated. The prevalence of ESBL-*E. coli* and -*K. pneumoniae* in BTM was 21.2% and 6.1%, respectively. ESBL-*E. coli* were detected in 41.2% of the study farms. Seven of the ESBL-*E. coli* isolates were multidrug resistant (MDR). The two ESBL-producing *K. pneumoniae* isolates were resistant to ceftriaxone. Seven ESBL-*E. coli* strains carry the *bla*_CTX-M_ gene, and five of them co-harbored *bla*_TEM-1_. ESBL-*E. coli* co-harbored *bla*_CTX-M_ with other resistance genes, including *qnr*B*19*, *tet*(A), *aad*A1, *aph(3’’)*-Ib, *aph(6)*-Id), *floR*, *sul2*, and chromosomal mutations (*gyrA, gyrB, parC, parE, and pmrB*). Most *E. coli* resistance genes were associated with mobile genetic elements, mainly plasmids. Six sequence types (STs) of *E. coli* were detected. All ESBL-*E. coli* were predicted to be pathogenic to humans. Four STs (three ST10 and ST69) were high-risk clones of *E. coli*. Up to 40 virulence markers were detected in all *E. coli* isolates. One of the *K. pneumoniae* was ST867; the other was novel strain. *K. pneumoniae* isolates carried three types of beta-lactamase genes (*bla*_CTX-M_, *bla*_TEM-1_ and *bla*_SHV_). The novel *K. pneumoniae* ST also carried a novel IncFII(K) plasmid ST.

**Conclusion:**

Detection of high-risk clones of MDR ESBL-*E. coli* and ESBL-*K. pneumoniae* in BTM indicates that raw milk could be a reservoir of potentially zoonotic ESBL-*E. coli* and -*K. pneumoniae*.

## 1. Introduction

In the United States of America (U.S.), beta-lactam antibiotics, specifically ceftiofur, are frequently used for prophylactic and therapeutic purposes in dairy cattle farms (Food and Drug Administration (FDA), 2016; Redding et al., 2019; Gelalcha et al., 2021; Gelalcha and Kerro Dego, 2022). This heavy use can drive the emergence of extended-spectrum beta-lactamases (ESBL) producing bacteria, which confers resistance to beta-lactam antibiotics, including third-generation cephalosporins (3GC) (Afema et al., 2018). As a result, multi-drug resistant (MDR) ESBL-producing bacteria such as *E. coli* and *Klebsiella* spp., the main trafficators of ESBL genes (*bla*_CTX-M_, *bla*_TEM,_ and *bla*_SHV_), are becoming increasingly prevalent in the U.S. dairy farms (Afema et al., 2018; Gelalcha et al., 2022; Gelalcha and Kerro Dego, 2022). These ESBL-*E. coli* and *Klebsiella* spp. can enter the bulk tank milk from subclinically infected mammary glands, contaminated udders, milking machines, and the dairy farm environment (Straley et al., 2006). The ESBL-*E. coli* and *Klebsiella* spp. in dairy farms may spill over to humans indirectly by consuming contaminated, unprocessed dairy products such as raw milk (Christy et al., 2018).

In recent years, there has been a growing trend toward more natural products and the promotion of the “probiotic effects” (i.e., naturally occurring beneficial bacteria or microorganisms in milk that promote or improve health when consumed) of raw milk, leading to an increase in raw milk consumption in the U.S. Some reports estimated that more than 3% of the population in the U.S. consumes raw milk, and at least 30 states in the U.S. legally accepted the sale of unpasteurized milk for human consumption (Whitehead and Lake, 2018). This highlights the increased risk of infection with MDR bacteria that are difficult to treat.

Consuming raw dairy products can increase the risk of ESBL-producing bacteria, such as *E. coli* and *Klebsiella* spp., spreading to humans through the food chain, especially milk, and colonizing the gastrointestinal tract (Liu et al., 2020). These bacteria can spread ESBL genes commonly associated with the mobile genetic element (MGEs) to other more harmful human pathogenic bacteria via horizontal gene transfer, creating a reservoir of antibiotic-resistant bacteria that threatens public health (Afema et al., 2018; Kuehn, 2018). In addition to transferring ESBL genes, some *E. coli* and *Klebsiella pneumoniae* strains are highly pathogenic and can cause various diseases in humans on their own (Decano et al., 2021).

Some studies reported a possible co-occurrence of ESBL genes, other multiple resistance genes, and virulence in *E. coli* and *K. pneumoniae* isolates from dairy cattle and other farm environmental samples (Hennequin and Robin, 2016; Ferreira et al., 2019; Lam et al., 2019; Yang et al., 2019). This further increases public health risks if these bacteria end up in raw milk meant for human consumption.

Although there is limited evidence linking dairy products as a source of ESBL producing-*Enterobacteriaceae* for humans, the high prevalence of ESBL-*Enterobacteriaceae* in dairy farms combined with the growing demand for unpasteurized milk suggests that raw milk may serve as a potential source and exit route for ESBL-*E. coli* and -*Klebsiella* spp. (Nobrega et al., 2018) from dairy farms to humans. This could be a potential source of the rising incidence of community-associated ESBL-*Enterobacteriaceae* infections in the U.S. (CDC, 2019).

Previous studies on raw milk in the U.S. were focused on microbiological identification of the commonly reported milk borne pathogens, such as *Staphylococcus aureus, Salmonella* spp.*, Listeria monocytogenes,* and *E. coli* O157:H7 (Karns et al., 2007; Langer et al., 2012; Mungai and Gould, 2015; Liu et al., 2020; Gelalcha and Kerro Dego, 2022). Few other studies in Asia (Tark et al., 2017; Liu et al., 2020) and Europe (Filioussis et al., 2020) reported the detection of ESBL-*E. coli* from mastitic milk samples. A 12-year retrospective study on mastitic milk in Wisconsin also identified a significant number of *Klebsiella* spp. resistant to ceftiofur, a 3GC whose resistance is mainly mediated by the production of ESBL genes (Fuenzalida et al., 2021). This study was solely based on phenotypic detection of resistance and did not examine the genetic characteristic of the isolates. Another recent research on *Klebsiella* isolates from mastitic milk reported several resistance genes that mediate resistance to critically important antibiotics (CIAs), including ESBL genes (Zheng et al., 2022). Milk from cows with mastitis is usually discarded and not used for human consumption. Thus, detecting ESBL-producing bacteria in such milk may not entirely indicate public health risks posed by raw milk consumption.

In our recent study, we detected ESBL-*E. coli* co-carrying resistance genes to multiple classes of antibiotics in bulk tank milk obtained from dairy farms (Gelalcha et al., 2022). In that study, we tested a limited number of farms and detected ESBL-*E. coli* using PCR. However, PCR is less informative (i.e., it does not offer a comprehensive view of the entire genome) only provides information about the specific gene(s); it cannot identify novel variants of ESBL genes, and it does not provide information about the genetic context or neighboring genes. WGS provides a complete genomic profile allowing the detection of not only ESBL genes and their variants but also other resistance genes, novel or unknown variants of resistance genes, mutations, mobile genetic elements and more complete understanding of genetic diversity. It also helps to identify genetic similarities and differences between bacterial strains and possible sources of milk contamination. Furthermore, WGS provides information about the genomic context of the ESBL genes (e.g., their association with mobile genetic elements), which is crucial in understanding how they are disseminated and how they might be linked to other genetic elements.

To the best of our knowledge, published reports on the detection of ESBL-*K. pneumoniae* from BTM in the U.S. and elsewhere are unavailable and that of ESBL-*E. coli* is limited. Thus, further studies using high-resolution molecular tools such as WGS are necessary to understand the status and distribution of ESBL-*E. coli and -Klebsiella* spp. in dairy farms and the risk for human health. This information can help increase public awareness about the potential health risks of raw milk consumption. Therefore, the objective of this study was to determine the prevalence of ESBL-*E. coli and -Klebsiella* spp. in bulk tank milk and characterize their antimicrobial resistance phenotypes, genotypes, virulence genes, and potential pathogenicity for humans.

## 2. Materials and methods

### 2.1. Isolation and identification of ESBL-*Escherichia coli* and -*Klebsiella pneumoniae* from bulk tank milk

Approximately 40 mL of bulk tank milk samples were collected into sterile 50 mL falcon tubes (mostly in duplicates to increase chance of detection of the bacteria) from 17 farms. The samples were transported to the laboratory in a cooler. A 20 mL of milk samples were suspended in 80 mL TSB-PO4 (MG Scientific, Pleasant Prairie, WI, United States). A 100 μL of the suspension was streaked on CHROMagar ESBL plates (a differential and selective media; DRG International, Inc., Springfield, NJ, United States) to isolate ESBL-*E. coli* and -*Klebsiella* spp. Plates were aerobically incubated for 24 h at 37°C. From each plate with visible growth, two presumptive ESBL-*E. coli* (dark pink to reddish) and *Klebsiella* spp. (metallic blue) colonies were subcultured into new CHROMagar ESBL plates (DRG International Inc.) and incubated for 24 h at 37°C to obtain a pure culture. Up to two well-isolated colonies were selected and inoculated into 5 mL of Luria Bertani broth (LBB; Thermo Fischer Scientific, Waltham, MA, United States) and grown overnight at 37°C while shaking. A 0.5 mL overnight culture was added to a 2 mL serum block, mixed with an equal volume of 80% sterile glycerol, and stored at −80°C for further tests.

Presumptive *E. coli* and *Klebsiella* isolates stored at -80^o^c were thawed and plated on CHROMagar ESBL plates and incubated at 37°C for about 18 h. A single colony of *E. coli* and *Klebsiella* spp. was picked and subcultured on blood agar (Thermo Fischer Scientific) at 37°C for about 18 h. Subsequently, *E. coli* and *Klebsiella* spp. was identified using matrix-assisted laser desorption ionization time-of-flight mass spectrometry (MALDI-TOF MS) as described by the manufacturer (Bruker Daltonics, Billerica, MA, United States) at the University of Tennessee, College of Veterinary Medicine, Diagnostic Bacteriology and Mycology Lab. The samples were prepared using a formic acid (FA) extraction method (Doern and Butler-Wu, 2016). The MALDI Biotyper library version 11.0 and MALDI Biotyper software version 4.1.100 (Bruker Daltonics) were used for this analysis. For identifying *E. coli* and *Klebsiella* spp, the manufacturer-recommended cutoff score values of 2.0 to 3.00, which is considered high-confidence identification, were used.

### 2.2. Antimicrobial susceptibility testing

Antimicrobial susceptibility testing (AST) was performed on all MALDI-TOF MS-confirmed *E. coli and Klebsiella* spp. isolates against 14 antimicrobials representing 10 classes of antimicrobials using the broth microdilution method. Commercially available 96-well microtiter plates containing the 14 antimicrobial panels (Sensitire™ CMV4AGNF; Thermo Fisher Scientific) were used. The 14-antimicrobials include *β*-lactams (ampicillin, ceftriaxone, cefoxitin, amoxicillin-clavulanic acid, meropenem), Aminoglycosides (gentamicin and streptomycin), sulfonamides (sulfisoxazole, trimethoprim/sulfamethoxazole), macrolides (azithromycin), phenicols (chloramphenicol), quinolones (ciprofloxacin and nalidixic acid), and tetracyclines. The minimum inhibitory concentrations (MIC) of the 14 antimicrobials were determined following the manufacturer’s recommended protocol and following CLSI M100 Clinical Laboratory Standards Institute guidelines (CLSI M100: *Enterobacteriaceae*; Clinical and Laboratory Standards Institute (CLSI), 2022).

### 2.3. DNA extraction

The MALDI-TOF MS confirmed *E. coli* and *Klebsiella* isolates stored at -80°C were thawed and plated on CHROMagar ESBL plates (DRG International Inc.). After overnight incubation, 2–3 colonies were picked with a sterile inoculating wire loop, subcultured into 5 mL LBB (Thermo Fischer Scientific) and incubated at 37°C for 18–24 h. Bacterial DNA was extracted using the Qiagen MagAttract HMW DNA kit (Qiagen, Germantown, MD, United States) following the manufacturer’s procedure with some modifications. Briefly, 2–3 bacterial colonies were taken from a pure overnight culture using a sterile wire loop and dissolved into 5 mL LBB (Thermo Fisher Scientific). The broth was incubated overnight at 37°C, and 2 mL was transferred into the 2 mL microcentrifuge tube. The tube was placed in a centrifuge (Thermo Fisher Scientific) and centrifuged at 5,000 × g for 10 min. Then, the supernatant was discarded, and the pellet was suspended in a lysis solution, placed in shaker-incubator series (VWR International, Philadelphia, PA, United States), and shaken at 500 rpm for 1.25 h at 57°C. After adding 4 μL RNase and incubating for 2 min at room temperature, 15 μL magnetic beads and 280 μL binding buffer (Buffer MB) were added to the lysate solution and pulse vortexed and placed in a MagAttract magnetic rack (Qiagen) and shaken in a shaker-incubator serious (VWR International) at 500 rpm for 15 min. The Magnetic Rack was placed on the magnetic base so that the magnetic beads, along with the DNA, would get separated by sticking to the side of the tube toward the magnetic base, and the supernatant was removed. The magnetic beads (together with the DNA samples attached) were washed twice each by Buffer MW1 and Buffer PE (Qiagen). The magnetic beads were rinsed twice with distilled water while the tube holder was on the magnetic base, and the beads were fixed to the walls of the microcentrifuge tube. Then, the tube holder of the Magnetic Rack was removed from its magnetic base, and 150 μL Buffer AE (elution buffer; Qiagen) was added. The tube holder of the Magnetic Rack was placed in incubator-shaker series (VWR International) and incubated at room temperature for 15 min at 500 rpm. The tube holder of the Magnetic Rack was placed on its magnetic base to separate the beads from the supernatant. Finally, the supernatant (elute) that was expected to contain high-molecular-weight DNA was transferred into a new sample tube. DNA concentration and purity were measured by a Nanodrop 2000 spectrophotometer (Thermo Fisher Scientific).

### 2.4. Library preparation and whole-genome sequencing of ESBL-*Escherichia coli* and -*Klebsiella pneumoniae*

Genomic libraries were prepared using the Nextera XT DNA library prep kit (Illumina Corporation, San Diego, CA, United States) following the manufacturer’s instructions. Final concentrations were confirmed on a Qubit fluorometer (Thermo Fischer Scientific). Some libraries checked for size and concentration on a high-sensitivity Bioanalyzer (Agilent Technologies, Santa Clara, CA, United States). Samples were normalized and pooled, and a final quality confirmation analysis was performed on a Bioanalyzer (Agilent Technologies). The pooled samples were diluted to a final loading concentration of 0.5 nM and combined with 5% PhiX control (Illumina Corporation). The sample pool and PhiX were then loaded on one lane of an SP flow cell reading 250 nucleotides paired-end (2× 250) on the Illumina NovaSeq (Illumina Corporation). The DNA library was sequenced at the University of Tennessee Genomics Core (Knoxville, TN, United States).

### 2.5. Quality control, assembly, and analyses of whole genome sequence data

Samples were demultiplexed, and quality assessment was done via FastQC v0.11.9 (FastQC, 2010) and multiQC v1.14 (Ewels et al., 2016). Adapter sequence removal, filtering, and trimming of low-quality raw reads were done using Trimmomatic v0.39 (Bolger et al., 2014; LEADING:5 TRAILING:5 SLIDINGWINDOW:5:20 MINLEN:30). Preprocessed reads of each isolate were *de novo* assembled using SPAdes genome assembler v3.15.5 (Bankevich et al., 2012) with default parameters. PlasmidSPAdes modes were used for the *de novo* assembly of the “plasmids only” for downstream analysis.

To further confirm the species of bacteria identified by MALDI-TOF MS, KmerFinder 3.2^1^ was used to predict bacterial species using a fast K-mer algorithm from the assembled genome (Hasman et al., 2014; Larsen et al., 2014; Clausen et al., 2018). The WGS data were used to identify variants of ESBL genes and detect virtually all known antibiotic resistance genes (ARGs) or mutations co-harbored with ESBL genes. To determine the presence of acquired antibiotic resistance genes and chromosomal point mutations in assembled genomes of the isolates, ResFinder v4, online bioinformatics tools at the Center for Genomic Epidemiology^2^ (Zankari et al., 2017; Bortolaia et al., 2020) and Comprehensive Antimicrobial Resistance Database (CARD^3^; Alcock et al., 2020) were employed using *E. coli* and *Klebsiella* spp. specific panels. ESBL-encoding genes and other resistance determinants were considered present when at least 60% of the length of the best-matching gene in the ResFinder database was covered with sequence identity greater than or equal to 95% (Feldgarden et al., 2019).

To identify plasmids and other mobile genetic elements (MGE) associated with ARGs, draft assemblies of ESBL-*E. coli* and *Klebsiella* spp. genome was analyzed using PlasmidFinder 2.1^4^ and MGE Finder v1.0.3.^5^ The PlasmidFinder detects plasmids and assigns them to incompatibility groups (Camacho et al., 2009; Carattoli et al., 2014). At the same time, MGE Finder was used to identify plasmids and other MGEs, such as transposon (Tn) and Insertion Sequences (IS) associated with ARGs and virulence genes (Camacho et al., 2009; Zankari et al., 2012; Joensen et al., 2014). The MGEs were identified with a minimum 98% sequence identity threshold with the closest-matching hits and minimum 90% coverage (Carattoli et al., 2014). The virulence gene associated with MGE in each genome was identified with 98% sequence identity and a minimum of 95% coverage using virulenceFinder. The virulence markers in the isolates were identified using virulenceFinder^6^ as described before (Joensen et al., 2014; Malberg Tetzschner et al., 2020).

To investigate genetic relatedness, the WGS of isolates were analyzed using *in silico* sequence serotyping (*E. coli* only), multilocus sequence types (MLST), and Core genome Multi Locus Sequence Type (cgMLST) for both *E. coli* and *Klebsiella pneumoniae*. Multilocus sequence types (MLST) of *E. coli* were identified according to the Achtman seven-locus MLST approach, which is based on sequences of seven housekeeping genes (*adk*, *fum*C, *gyr*B, *icd*, *mdh*, *pur*A, and *rec*A) which are known to present in all strains of *E. coli* and are relatively stable in terms of their sequence (Wirth et al., 2006; Larsen et al., 2012; Clermont et al., 2015). For *Klebsiella* spp., MLST was identified based on seven housekeeping genes (*gapA, InfB, mdh, pgi, phoE, rpoB, and tonB*) described in other studies (Diancourt et al., 2005; Larsen et al., 2012). The serotype of *E. coli* isolates was predicted from the draft assembly using SerotypeFinder 2.0.1^7^ with 95% threshold identity and 80% minimum length (Camacho et al., 2009; Clausen et al., 2018). The MLST of both species of bacteria was predicted from the assembled draft genome using MLST 2.0 2.0^8^ as described in Bartual et al. (2005), Lemee and Pons (2010), and Larsen et al. (2012). The assignment of cgMLST of *E. coli* was done using cgMLSTFinder 1.2^9^ as described in previous studies (Clausen et al., 2018; Zhou et al., 2020). The pathogenicity of both species of bacteria was predicted from their genome based on gene families that correlate with pathogenicity using PathogenFinder 1.1^10^ as described in Cosentino et al. (2013). The pathogen finder tool evaluates the disease-causing potential of a bacterial strain. We begin by uploading the draft sequence of the *E. coli* genomes onto the web-based platform. The tool then compares the proteins encoded by the *E. coli* strains to a database containing groups of proteins or protein families that were associated with pathogenic or non-pathogenic organisms. Initially, the tool clusters these proteins, identifying significant clusters where most proteins originate from either pathogens or non-pathogens. Subsequently, protein families are labeled as pathogenic or non-pathogenic, and a weight (Z-score) is calculated for each based on matched protein families. Finally, the tool predicts whether the input organism is human pathogenic or not based on the associated probability value from the prediction (Cosentino et al., 2013). Plasmid Multilocus Sequence Typing (pMLST) was carried out on draft assembled plasmids on incompatibility groups for which a pMLST scheme is available using^11^ as described in Carattoli et al. (2014).

## 3. Results

### 3.1. Prevalence and antimicrobial resistance profiles of ESBL-*Escherichia coli* and -*Klebsiella pneumoniae* in bulk tank milk

A total of eight ESBL-*E. coli* and two ESBL-*K. pneumoniae* strains were isolated from 33 milk samples collected from 17 farms. The isolates were detected from nine farms; one *E. coli* and *K. pneumoniae* were isolated from the same farm, whereas the others were detected from 8 different farms. From the eight *E. coli* isolates identified with ESBL phenotype, one isolate, CD-M, was later confirmed to be susceptible to all tested antibiotics. Thus, the sample level prevalence of ESBL*-E. coli* was 21.2% (7/33), whereas that of *Klebsiella pneumoniae* was 6.1% (2/33). At the farm level, ESBL-*E. coli* and -*K. pneumoniae* prevalence was 41.2% (7/17) and 11.8% (2/17), respectively.

All eight *E. coli* isolates identified in this study were tested for antimicrobial susceptibility against 14 antimicrobial agents. All *E. coli* isolates with ESBL phenotype (*n* = 7) were co-resistant to ampicillin, ceftriaxone, and tetracycline, and all of them were multidrug resistant (resistant to three or more antimicrobial classes). More than half of the ESBL-*E. coli* isolates (*n* = 4) were phenotypically resistant to five or more antimicrobials, including those considered the highest priority critically important classes (HPCI) such as third generation cephalosporins and fluoroquinolones. The ESBL phenotype co-existed with other resistant phenotypes to CIAs, such as ceftriaxone, streptomycin, and fluoroquinolones (nalidixic acid or ciprofloxacin) in four *E. coli* strains. One *E. coli* isolates (AG-M) was resistant to nine antimicrobial agents belonging to 7 classes. All *E. coli* isolates were susceptible to meropenem, cefoxitin, and amoxicillin-clavulanic acid (Table 1).

**Table 1:** Antimicrobial resistance phenotypes and mobile genetic elements associated with resistance genes in ESBL-*Escherichia coli.*

Isolate	Antimicrobial resistance									MGE* linked to resistance genes
	Phonotypes	Genes								
		βRG	FQRG	ARG	MRG	PRG	FARG	TRG	PM	
AG-M	AXO, AMP, AZI, CIP, NAL, STR, CHL, STX, TET	*bla*_CTX-M-32_^a^	*qnrB19*^b^	*aadA1*	*mph*(A)^c^	*floR*^d^	*sul1*, *dfrA1*	*tet*(A)	*gyrA parC*	IS630^a^
										Col440I^b^
										IncR^c^
										IS6^d^
RM-M	AXO, AMP, FIS, TET	*bla*_CTX-M-32_e, *bla*_TEM-1A_^f^	*qnrB19*^g^	*aph*(3″)-Ib^f^, *aph*(6)-Id ^f^	-	*flo*R^f^	*sul2*^f^	*tet*(A)^f^	*gyrA*	IS5^e^
									*parE*	IS91^f^
									*parC*	Col440I^g^
DM-M	AXO, AMP, CIP, NAL, GEN, STR, CHL, TET	*bla*_CTX-M-27_^h^_,_ *bla*_TEM-1c_^i^	*qnrB19* ^j^	-	-	-	*sul2*	*tet*(A)^i^	*gyrA*, *gyrB*, *parC*, *pmrB*, ampC^*^	IncFII^h^, IS6^h^
										Tn1^i^
										IncFII(pCoo)^i^, Col440I^j^
ST-M	AXO, AMP, STR, CHL, FIS, TET,	*bla*_CTX-M-32_, *bla*_TEM-1A_^j^	*qnrB19*^k^	*aph*(3″)-Ib^j^, *aph*(6)-Id ^j^	-	*floR*^l^	*sul2*^j^	*tet*(A)	*parC*	Tn3^j^, Col440I^k,^ IncFIA(HI1)^l^
									*pmrB*	
DA-M	AXO, AMP, TET	*bla*_CTX-M-65_^m^	-	-	-		*sul2*^m^	*tet*(A)	*parC*, ampC^*^	IS6 ^m^
RC-M	AXO, AMP, TET	*bla*_CTX-M-32_, *bla*_TEM-1Bn_	*qnrB19* ^o^	-	-	-	-	*tet*(A)^n^	*parC*, *parE*, *gyrA*, *pmrB*	Tn1721^n^, Col440I^o^
BET-M	AXO, AMP, CIP, NAL, STR, CHL, FIS, TET	*bla*_CTX-M-32_^p^, *bla*_TEM-1A_^q^	*qnrB19*	*aph*(3″)-Ib^q^, *aph*(6)-Id^q^	-	*floR* ^q^	*sul2* ^q^	*tet*(A) ^q^	*gyrA*	IS5^p^, IS91^q^
									*parE*	
									*parC*	
CD-M	-	-	-	-	-	-	-	-	*parC*, *ampC* ^*^ *pmrB*	-

*MGE, mobile genetic elements. ^a–q^Resistance genes with the same superscript letters indicate they are co-located on the same contigs together with mobile genetic elements with the same superscripts. ^*:^ Mutation is on the promoter regions of *amp*C. AXO, ceftriaxone; FOX, cefoxitin; AUG2, amoxicillin-clavulanic acid; AMP, ampicillin; MERO, meropenem; AZM, azithromycin; CIP, ciprofloxacin; NAL, nalidixic acid; CHL, chloramphenicol; GEN, gentamicin; STR, streptomycin; TET, tetracycline; FIS, sulfisoxazole; STX, trimethoprim-sulfamethoxazole; βRG, beta-Lactamases resistance genes; FQRG, Fluoroquinolones resistance genes; ARG, aminoglycosides resistance genes; MRG, macrolides resistance genes; PRG, phenicol resistance genes; FARG, folate antagonist resistance genes; TRG, tetracycline resistance genes; PM, point mutation.

### 3.2. Resistance genes and their associations with mobile genetic elements in *Escherichia coli*

The eight bacterial strains initially identified as *E. coli* by MALDI-TOF MS were indeed confirmed to be *E. coli* through WGS. One of these is ESBL-*E. coli* isolates (CD-M) were found to have no resistance genes to beta-lactam antibiotics or many other classes of antibiotics. Genome sequence analysis did not find the presence of ESBL or other ARGs in *E. coli* isolates that were phenotypically susceptible to all tested antibiotics.

All seven ESBL-*E. coli* strains carry the *bla*_CTX-M_ variant and five isolates co-carry *bla*_TEM-1_. Among ESBL genes, *bla*_CTX-M-32_ was a common variant and was detected in five of the isolates. All ESBL-*E. coli* strains co-harbored *bla*_CTX-M_ with plasmid-mediated quinolone resistance gene (*qnrB19*), and a tetracycline resistance gene, *tet*(A).

Resistance genes to aminoglycosides [*aad*A1 or *aph*(3″)-Ib, *aph* (6)-Id], quinolones (plasmid-mediated and point mutations), phenicol (*floR*), and sulphonamides (*sul2*) were concurrently detected in four ESBL-*E. coli* strains that harbored the *bla*_CTX-M-32_ variant. The macrolide phosphotransferases [*mph*(A)], aminoglycoside nucleotidyltransferase (*aad*A1), dihydrofolate reductase (*dfrA*), and dihydropteroate synthase (*sul1*) resistance genes were detected in a single isolate, AG-M. As expected, *bla*_AmpCs_ and carbapenemase-associated genes were not identified.

Mutation in the *amp*C gene promoter region was detected in three isolates (CD-M, DA-M, and DC-M) but was not expressed. Double or triple mutations in Quinolone-Resistance Determining Regions (QRDRs) of *gyrA*, *gyrB*, *par*C, and *parE* were detected in six isolates. These multiple chromosomal mutations were (*gyrA*-S83L, D87Y, D741E, D87G, D87N, D678E, Q454H, and D741E; *par*C-E62K or S80I; *parE*-L416F and *gyrB*-Y184D). A point mutation that potentially confers resistance to colistin, *pmrB*, was detected in four of the isolates. For one isolate (DM-M), the genotypes that mediate the phenotypic resistance, such as aminoglycosides and chloramphenicol, were not detected in the Center of Genomic Epidemiology database (see text footnote 2). But we identified *marA* and *evgA*, genes that encode efflux pumps known to mediate resistance to several classes of antibiotics, including aminoglycosides and chloramphenicol in comprehensive Antibiotic Resistance Database (CARD) (see text footnote 3).

A quinolone resistance gene, *qnrB19*, was consistently associated with Col440I plasmid replicon types in six ESBL-*E. coli* isolates. All except one *bla*_CTX-M_ gene were associated with MGEs alone or with other resistance genes. In some of the ESBL-*E. coli* isolates, multiple resistance genes are co-located on the same contigs associated with the same or different MGEs (Table 1). In two of the ESBL-*E. coli* strains, BET-M, and RM-M, six other resistance genes (*aph*(3″)-Ib, *aph*(6)-Id, *tet*(A), *floR*, *sul2*, *bla*_TEM-1A_), mediating resistance to five different classes of antibiotics were all found on the same contigs associated with IS91. All except one *bla*_CTX-M_ and almost all other co-harbored acquired ARGs were associated with MGEs such as plasmids, Insertion sequences (IS), or transposons (Tn) on the same genetic fragment.

### 3.3. Genetic diversity, plasmid types, and pathogenomics of ESBL*-Escherichia coli* isolates

The *E. coli* isolates were assigned to seven distinct serotypes, six multilocus sequence types (STs), and eight-core genome sequence types (cgSTs) based on *in silico* sequence typing of the WGS data. Two isolates from different farms, RM-M and BET-M, had the same serotype (O101:H9) and sequence type (ST10). All eight strains were distinct according to their core genome sequences, based on comparing nearly all the genes shared by *E. coli*. The ST10 type was relatively common and found in three farms. Three isolates, AG-M, ST-M, and DA-M had identical genotypes (ST and resistance gene pattern) as *E. coli* isolates from fecal samples collected from dairy cattle in the same farms where BTM was also obtained (unpublished data).

PlasmidFinder identified 13 different plasmid replicon types from the WGS data of the seven ESBL-*E. coli* isolates. Each ESBL-*E. coli* isolate had at least two plasmid replicon types. The IncF family plasmids (FII, FIA, and FIB replicon types) were common and found in four ESBL-*E. coli* isolates. Colcinogenic plasmids (Col440I) were the most frequent and detected in all seven ESBL-*E. coli* isolates. The ST10 type carried the largest number (6 to 8) and types of plasmids. Two ST10 and ST69 had epidemic resistance plasmids of the IncF family. No plasmid was detected in the WGS of one *E. coli* isolate, CD-M, which was susceptible to all tested antibiotics.

Several virulence genes were detected in all eight *E. coli* isolates. Each isolate had multiple virulence genes involved in adhesion (adhesin), invasion (invasins for bacterial fitness), and effectors or toxins (Table 2). The highest number of virulence genes (Zankari et al., 2017) were detected in the ST69 isolate, and the lowest number (Christy et al., 2018) in the isolate ST5891 susceptible to all tested antibiotics. Widespread virulence genes, such as *asl*A (arylsulfatase-like gene)*, fim*H (type1 fimbriae)*, yeh, tre*C (tellurium ion resistance protein encoding gene)*, gad* (glutamate decarboxylase encoding gene)*, csg*A (curli fimbriae gene)*, ter*M*, hyl*F (hemolysin)*, iss* (increased serum survival protein encoding gene), and *tra*T (outer membrane protein responsible for complement resistance), were found in the *E. coli* genomes. No hits for the Shiga toxin-producing gene were detected in the WGS of the isolates. The draft sequences of all *E. coli* isolates were submitted to PathogenFinder, (see text footnote 10) and all were predicted as human pathogens with a probability greater than 0.92.

**Table 2:** ESBL-*Escherichia coli* genetic diversity and pathogenomics.

Isolate	Serotype	MLST^a^	CgMLST^b^	Virulence genes and their roles			Predicted Probability of pathogenicity to human
				Colonization (adhesins)	Fitness (invasins)	Effectors (toxins)	
AG-M	045:H25	2325	133328	*fimH*, *yeh* B, C & D, *nlpI*	*cea*, *sitA*, *terC*, *iss*, *csgA*, *gad*	*hlyE*	0.935
RM-M	O101:H9	10	34072	*fimH*, *fdeC*, *yeh* A, B, C &D, *nIpI*	*iss*, *terC*, *csgA aslA*, *hha*	*hlyE*	0.921
DC-M	O17/O44:H18	69	33877	*afaA*, B, C, & D; *yeh* A, B, C & D; *fae* C, D, F, I &J; *aalF lpfA*, *fimH*, *fdeC*, *air*(*eaeX*), *eila*, *nlpI*	*traT*, *traJ*, *sitA*, B, & C; *iss*, *terC*, *chuA ompT*, *csgA*, *cvaC*, *aslA*, *gad*, *kpsMIII*_K98, *kpsE*, *mchF*, *hha*	*hlyE*, *astA*, *espP*	0.933
ST-M	09:H30	540	160799	*fdeC*, *fimH*, *lpfA yeh* A, B, C &D, *nlpI*	*aslA*, *terC*, *iss*, *gad*, *csgA*, *hha*	*hlyE*	0.935
DA-M	O36:H36	10	115817	*yeh* A, B, C & D; *fde*C, *fimH*, *nlpI*	*csgA*, *terC*, *traT*, *fyuA*, *aslA*, *irp2*	*hlyE*	0.934
RC-M	O142:H29	5728	5861	*yehB*, C &D; *fdeC*, *nlpI*	*iss*, *csgA*, *terC*, *aslA*, *gad*, *hha*	*hlyE*	
BET-M	O101:H9	10	34081	*fdeC*, *fimH*, *yeh* A, B, C &D, *nlpI*,	*aslA*, *terC*, *iss*, and *csgA*, *gad*, *hha*	*hlyE*	0.925
CD-M	O112ab:H19	5891	197619	*yehA*, B, C, &D, *lpfA*, *fimH*, *fdeC*,	*terC*, *csgA*, gad	*hlyE*	0.944

^a^MLST: Multilocus Sequence Type; CgMLST^b^: core genome Multilocus Sequence Type.

### 3.4. Co-localization of virulence genes and mobile genetic elements

Several virulence genes were detected on ESBL*-E. coli* were co-located on the same contigs with the MGEs (plasmids, insertion sequences, and transposons). In addition, multiple virulence genes were often co-located with or on the same MGEs. In some instances, copies of the same virulence genes were associated with different MGEs (e.g., plasmids and transposon). Most virulence genes related to MGE are those involved in the invasion and adhesion of mammalian cells. Only two isolates have effector (protease) encoding genes associated with MGEs. Table 3 showed several virulence genes associated with one or more MGEs.

**Table 3:** Virulence genes and their association with mobile genetic elements.

Isolate	Virulence genes and their role		
	Colonization (adhesins)	Fitness (invasins) and Effectors (toxins)	The mobile genetic element associated with the virulence genes
AG-M	*fimH*, *yeh* B, C &D,	*cea*, *sitA*, *terC*, *iss*, *csgA*, *gad*	None
RM-M		*iss*	(IS1, IS630)
DC-M	(*afaA*-D, & *terC*)^a^ *ompT*^b^ (*yeh* A, B, C &D)^c^	*traT*^d^, (*terC*, *kps*MIII_K98, *kpsE* & *nlpI*)^e^, *mchF*^f^, *gad*^h^, *espP*^g^	IS630^a^, IS1^b^, IS605^c^, (Tn1and IncFII(pCoo))^d^, IS5^e^, IncFIB(AP001918)^f^, (IS30 and IS1)^g^, (IS30 and IS1)^h^
ST-M	(*fdeC* & *iss*)^i^	*gad* ^j^, (*nIpI* & *terC*)^k^	IS1^i^, IS150^j^, IS630^k^
DA-M	*fdeC*^l^, *fimH*^m^,	*terC*^n^	(IS1, IS630)^l^, IS903^m^, (IS51&IS1)^n^
RC-M		*terC*^o^, *gad*^p^, (*hlyE* & *gad*)^q^, *aslA*^r^	IS630^o^, Tn1^p^, (IS605, IS3& IS4)^q^, IS3^r^
BET-M	(*fdeC* & *iss*)^s^	*terC*^t^, *gad*^u^,	(IS1 & IS630)^s^, (IncHI2A and IncHI2)^t^, (IS150 and IS1)^u^
CD-M	(*yehA*, B, C, & D)^v^, *fdeC*^w^	*terC*^x^	(IS3, IS630)^v^, (IS1 and IS630)^w^, IS630^x^

^a–x^virulence genes with the same superscript indicate they are co-located on the same contigs together with mobile genetic elements bearing the same superscripts. Those virulence genes in the same parentheses are co-located with Mobile genetic element/s having the same superscript.

### 3.5. Resistance phenotypes and genotypes in ESBL*-Klebsiella pneumoniae*

The two *K. pneumoniae* isolates were resistant to ceftriaxone and ampicillin. In addition to the two antibiotics, one of the isolates, DM-MK, was resistant to sufisoxazole. Each *K. pneumoniae* isolate carried two distinct types and variants of ESBL-genes, the *bla*_CTX-M,_ and *bla*_SHV_.

The *K. pneumoniae* isolates contain a multidrug efflux pump encoding gene, *oqxAB*. Both *oqxB* and *oqxA* genes were found in one of the isolates, and this isolate was resistant to sufisoxazole. The other isolate only had the *oqxA* gene and was susceptible to the same antibiotic. Both isolates are considered genotypically multidrug resistant, carrying at least eight resistant determinants against multiple classes of antibiotics. However, the observed phenotypic resistance was low (Table 4).

**Table 4:** Phenotypic and genotypic resistance in ESBL-*Klebsiella pneumoniae* isolates.

Isolate	Resistance phenotype	Resistance genes by antibiotic class						MGE	PHP
		βRG	FQRG	ARG	MRG	MEP	FRG		
ST867	AXO	*bla*_CTX-M-27_^a^, *bla*_SHV-62_, *bla*_TEM-1B_^a^	*qnrB19*^b^	-	-	*oqxB*, *oqxA*	*fosA*	*IncFII*^a^ *Col440I*	*0.89*
	AMP								
	FIS								
Novel ST	AXO	*bla*_CTX-M-1_, *bla*_SHV-2_, *bla*_SHV-40_	*qnrB19^c^*	*aph*(6)-Id	*mph*(A)	*oqxA*	*fosA*	*^c^Col440I*	*0.88*
	AMP								

AXO, ceftriaxone; AMP, ampicillin; FIS, sulfisoxazole; βRG, beta-lactamases resistance genes; FQRG, Fluoroquinolones resistance genes; ARG, aminoglycosides resistance genes; MRG, macrolides resistance genes; MEP, multidrug efflux pump; FRG, Fosfomycin resistance genes; MGE, Mobile Genetic Element associated with resistance genes; PHP, probability of being a human pathogen.

### 3.6. Genetic diversity, plasmid types, and pathogenomics of *Klebsiella pneumoniae*

The WGS data were used for *in silico* sequence typing on the two *K. pneumoniae* isolates. One of the isolates (BM-MK) was assigned as ST867, while the remaining one was novel and could not be assigned. The ST867 is identical to three other STs identified from fecal samples from the same farm where BTM was obtained. Both *K. pneumoniae* obtained from BTM were identified as new serotypes of *K. pneumoniae*. Both strains contain IncF family plasmids with replicon types FII. The ST867 strain only had the FII replicon. The novel strain had seven plasmids, including two Col plasmids (Col440I and II), four epidemic resistance plasmids with IncF plasmid families [FII, FII(K), FIA, and FIB replicons], and one IncN plasmid, which is a broad host range plasmid. This study identified a new allele type for IncFIA (HI1) plasmid using MLST. The WGS data also revealed the presence of multiple virulence genes such as *fimH* (type 1fimbriae), *fyu*A (siderophore receptor), *itu*A (ferric aerobactin receptor), *tra*T (outer membrane protein complement resistance), and *mrk*A (type 3 Fimbrial Shaft) in ST867. More number of virulence genes were detected in the novel *K. pneumoniae* ST. The virulence genes detected in this ST include *fim*H, *tra*T*, fyu*A, *iut*A*, mrk*A*, Irp*2 (non-ribosomal peptide synthetase), *ccI* (Cloacin), *clpK1*(thermal stress survival ATPase). Both *K. pneumoniae* were predicted to be human pathogens with a probability greater than 0.82.

## 4. Discussion

This study found a high prevalence of ESBL-producing *E. coli* in bulk tank milk (BTM) from dairy farms showing the significant public health associated with raw milk consumption. ESBL-*E. coli* was found in the BTM of 41% of the farms, and 21.2% of the BTM samples tested positive for these bacteria. Additionally, ESBL-producing *K. pneumoniae* was detected in two BTM samples from two farms. Since we did not come across similar studies conducted in the U.S. or elsewhere, a comparison of prevalence was not possible in the current study. However, in our earlier study that used different screening techniques, ESBL-*E. coli* was detected in at least one of the BTM samples collected from four farms (Gelalcha et al., 2022). Similarly, a study conducted in Turkey on bulk tank milk, which involved an initial enrichment step, reported a comparable ESBL-*E. coli* prevalence of 22.6% (Kürekci et al., 2019).

Similarly, studies conducted on milk from cows with mastitis in New York (Yang et al., 2019), South Korea (Tark et al., 2017), Greece (Filioussis et al., 2020), and China (Liu et al., 2020) detected ESBL-*E. coli*. A comparison of the prevalence of ESBL-*E. coli* between the current study and these studies are not plausible as the sample source, and the initial screening methods used were different. This suggests the prevalence of ESBL-*E. coli* in BTM may be higher than previously thought, highlighting the need for continuous monitoring and evaluation of associated risk and developing innovative control tools at farm levels.

The study also indicated that all ESBL-*E. coli* were MDR, with most of them displaying concurrent resistance to five or more antibiotic classes. These findings are consistent with previous studies in animals and humans that showed ESBL-*Enterobacteriaceae* frequently showed co-resistance to multiple classes of antibiotics (Masse et al., 2021; Badr et al., 2022; Gelalcha et al., 2022; Gelalcha and Kerro Dego, 2022). Four ESBL-*E. coli* also showed concurrent resistance to CIAs, such as ceftriaxone, fluoroquinolone (ciprofloxacin and/or nalidixic acid), and aminoglycosides (streptomycin and/or gentamycin). This result also agrees with our recent study and several other studies that reported ESBL-producers frequently exhibit co-resistance to fluoroquinolones, aminoglycosides, chloramphenicol, and tetracycline (Bradford, 2001; Canton and Ruiz-Garbajosa, 2011; Timofte et al., 2014; Afema et al., 2018; Agga et al., 2022; Gelalcha et al., 2022; Gelalcha and Kerro Dego, 2022). However, the study farms did not report aminoglycosides, tetracyclines, and fluoroquinolones as frequently used antibiotics. In addition, fluoroquinolones and macrolide are not allowed to use in the U.S. dairy cattle of more than 20 months of age (FDA, 2023). Thus, the detection of fluoroquinolone resistance in half of the isolates and azithromycin resistance in one of the isolates from BTM pooled from adult cows may not be related to antibiotic use. Further investigation is needed to identify if the use of these antibiotics at a young age on calves has an impact during adult age or if ceftiofur uses in dairy cattle co-select for resistance determinants against these two CIAs, as previously hypothesized (Taylor et al., 2020). In addition, it is also vital to examine if resistance genes against these two classes of antibiotics are maintained through bacterial generation via MGEs that confer a fitness advantage to the host.

In line with this thought, WGS analysis of the isolates identified that *qnrB19* (a gene mediating resistance to ciprofloxacin) was consistently associated with Col440I plasmids. A previous study showed that ColE1-derived plasmids, including Col440I, contain genes that encode for bacteriocins, proteins that can kill other bacteria and provide a competitive advantage to the host bacterium (Riley and Gordon, 1992). This suggests that the association of *qnrB19* with Col440I might be responsible for its maintenance in the absence of selection pressure. A recent study in Germany also reported frequent association of *qnrB19* with Col440I plasmids in ESBL-producing *E. coli* from livestock (Juraschek et al., 2022).

All isolates were susceptible to meropenem, one of the last resort antibacterial agents whose use is typically reserved for severe infections caused by pathogenic members of ESBL-*Enterobacteriaceae* (Karaiskos and Giamarellou, 2020). This finding is not unexpected, as carbapenems are not approved for animal use in the U.S. due to the risk of promoting the emergence of carbapenemase-producing *Enterobacteriaceae*. Detection of ESBL-*E. coli* with phenotypic resistance to multiple classes of antibiotics, particularly to those CIAs, makes consuming raw milk a dangerous practice for public health as only limited therapeutic options are available to cure the infection caused by the bacteria with these phenotypes (WHO, 2019).

The WGS analysis showed that the observed phenotypic resistance patterns are generally congruent with genotypic results, as reported in a previous study suggesting that the genotypes of the bacteria are a reliable indicator of their antibiotic resistance (Tyson et al., 2015). The *bla*_CTX-M_ was the only ESBL-variants observed from all ESBL-*E. coli* isolates phenotypically resistant to ceftriaxone, a 3GC. This finding concurs with our previous study, where we reported *bla*_CTX-M_ as the only ESBL variant from *E. coli* isolates retrieved from a dairy farm environment with phenotypic resistance to cefotaxime, another 3GC (Gelalcha et al., 2022). Similarly, other studies in the U.S. and elsewhere also showed the predominance of the *bla*_CTX-M_ variant of ESBL over the others among the *Enterobacteriaceae* in humans and food animals, including dairy cattle (Canton et al., 2012; Peirano et al., 2012; Weissman et al., 2013; Davis et al., 2015; Bevan et al., 2017; Afema et al., 2018; Collis et al., 2019; Gelalcha and Kerro Dego, 2022). It is hypothesized that the frequent occurrence and the dominance of the *bla*_CTX-M_ variant over the others in ESBL-*E. coli* may be related to its low fitness cost to the host, successful dissemination through MGEs, and rapid response to beta-lactam antibiotics selection pressure (influence or changes in bacteria exerted by the use of beta-lactam antibiotics that enable bacteria to became resistant; D’Andrea and Pallecchi, 2013). As we already discussed in our published review (Gelalcha and Kerro Dego, 2022), further controlled study is needed to identify the cause of the spread and dominance of this variant of ESBL genes.

The *bla*_CTX-M-32_ was detected in five farms, whereas the remaining two, *bla*_CTX-M-27_ and *bla*_CTX-M − 65_, were obtained from ESBL-*E. coli* retrieved from two separate farms. These *bla*_CTX-M − 32_ variants were previously reported from ESBL-*E. coli* isolated from fecal samples in the U.S. dairy farms (Afema et al., 2018; Taylor et al., 2020; Carey et al., 2022). The frequent presence of the non-ESBL beta-lactamase gene (*bla*_TEM-1_) along with the ESBL gene in *E. coli* may aid in maintaining and transmitting one or both genes to future generations clonally and to other bacteria horizontally via MGEs.

The fact that all ESBL-*E. coli* strains co-harbored *bla*_CTX-M_ with *qnrB19* (plasmid-mediated quinolone resistance gene), and *tet*(A) concurs with the phenotypic observation. But despite the presence of *qnrB19*, no corresponding resistance to ciprofloxacin was observed in some isolates. This discrepancy may be due to a low or lack of gene expression in that isolate due to various factors, including lack of activation of regulatory genes as we did not find mutation in the genes. Four ESBL-*E. coli* strains, co-harbored *bla*_CTX-M-32_ with resistance genes to aminoglycosides (*aad*A1 or *aph*(3″)-Ib, *aph(6)*-Id), quinolones (*qnrB19*), and mutation in one or more of fluoroquinolone resistance determining regions (QRDRs), phenicol (*floR*), and sulfonamides (*sul2*). This observation is also consistent with the phenotypic result and previous molecular studies that detect frequent co-occurrence of ESBL genes with multiple other ARGs (Afema et al., 2018; Peirano and Pitout, 2019; Gelalcha et al., 2022).

It is noteworthy that strains of ESBL-*E. coli* from two farms have the same six antibiotic resistance genes co-located on the same contigs with IS91, a mobile genetic element. Multilocus Sequence Typing (MLST) results showed that both strains belong to the same Sequence Type (ST10). However, the cgMLST typing identified both isolates as distinct strains. The two strains carry the same mutation in QRDRs (*gyrA, par*C, and *parE*) but differ in the *pmrB* gene presence and their plasmid replicon types. The detection of the same ARG with a similar genetic arrangement on the identical STs of *E. coli* needs further investigation. Still, a possible explanation could be that the strains may have a common ancestry and may have spread from one farm to another, parallel evolution between the two strains with the ARGs they carry, or contamination in the laboratory. However, contamination is less likely as the samples were collected and processed at different times.

All but one of the *bla*_CTX-M_ genes and most other ARGs co-harbored by the ESBL-*E. coli* were associated with mobile genetic elements (MGEs), such as plasmids, insertion sequences (IS), or transposons (Tn). The presence of ESBL and non-ESBL beta-lactamase genes, along with multiple ARGs on MGEs, is consistent with findings from previous studies on ESBL-*E. coli* isolates from dairy cattle and humans (Poirel et al., 2005; Afema et al., 2018; Filioussis et al., 2020; Liu et al., 2020; Toth et al., 2020; Gelalcha and Kerro Dego, 2022). This suggests that MGEs may facilitate the spread of these ARGs. The presence of multiple ARGs located on the same MGEs could make the transfer of these genes to other bacteria of the same or different species easier. This could lead to the development of extensively drug-resistant bacteria, which may risk human health through indirect transmission (Long et al., 2019; Toth et al., 2020).

Some of the ESBL-*E. coli* strains, ST10 and ST69, reported in this study had the same STs as previously reported as “high-risk” clones. These two sequence types were identified globally as the most prevalent extra-intestinal pathogenic *E. coli* (ExPEc) lineages after ST131 (Manges et al., 2019). A study on clinical *E. coli* isolates from 1999 to 2017 in California also identified these two STs as the most common causes of urinary tract infection (Yamaji et al., 2018). The ST10 and ST69 carry IncFII and/or IncI1, plasmid families. This finding is consistent with previous studies that reported high-risk clones of *E. coli* are frequently harbored by easily transmissible narrow host range or epidemic plasmids such as IncF and/or IncI1 (Johnson et al., 2007; Carattoli, 2009; Afema et al., 2018).

The detection of triple mutations in the QRDR region in two of the STs, ST10, and ST69, along with other ARGs (*qnrB19, floR*, *tet*(A), and *sul2*), indicated a public health risk associated with these bacterial clones. The public health risk or hazard is the potential that multidrug-resistant pathogenic ESBL-*E. coli* and *Klebsiella* species may reach the public indirectly through food chain (consumption of raw milk) and cause life threatening infections. These STs have been previously reported in fecal samples from dairy cattle and other food-producing animals in the U.S. (Afema et al., 2018; Hayer et al., 2020). The ST69 carried *bla*_CTX-M-27_ and *bla*_TEM-1c_ on IncFII (F2:A-:B-), an epidemic resistance plasmids known by its ability to acquire resistance genes and easily and rapidly spreading within the same or related species of bacteria (Carattoli, 2009). This ability to acquire and spread resistance genes quickly increases the prevalence of ESBL-producing bacteria and the difficulty of treating infections and is a significant public health concern.

In this study, the *pmrB* gene, which is commonly associated with colistin resistance (Delannoy et al., 2017), was detected in four *E. coli* isolates, including in two pandemic clones. Detecting a gene associated with colistin resistance is a public health concern as colistin is considered a last-resort antibiotic for treating infections caused by ESBL- and carbapenemase-producing *Enterobacteriaceae* (Falagas et al., 2011; Bialvaei and Samadi, 2015). The current study did not test for phenotypic colistin resistance because the panel of antibiotics used did not include colistin. Thus, the colistin gene’s impact on colistin resistance needs further study, but its presence still raises significant health risks. Furthermore, detecting colistin resistance genes in ST10 and ST69, epidemic strains, highlights the high risk associated with consuming raw milk. The plasmid-mediated colistin resistance gene, mcr-1, was previously detected in *E. coli* at a very low level (0.1%–0.02%) in the U.S. (Meinersmann et al., 2017; Wang et al., 2020) but no previous report on *Enterobacteriaceae* with any colistin resistance gene from BTM. The MDR ESBL-producing strains are already challenging to treat, and the additional presence of colistin-resistance genes increases the risk of treatment failure (Binsker et al., 2022). Detection of colistin resistance determinant in BTM was unexpected. This is because colistin has never been marketed for use in the U.S. dairy farms due to concerns about potential antibiotic resistance and to preserve it for human medicine as it is considered a last-resort antibiotic.^15^

All ESBL-*E. coli* isolates in this study carried several virulence genes exhibiting pathogenic properties (adhesion, invasion, and toxin production) in their mammalian host. The most frequently detected virulence genes in ESBL-*E. coli* were *fim*H, *csg*A, *yehA*-D, and *fde*C for adhesion; *iss*, *asl*A, *gad*, and *tra*T and *sit*A for bacterial replication and invasion; and *hyl*F for toxin secretion (Kaper et al., 2004; Paniagua-Contreras et al., 2017; Najafi et al., 2019; Abd El-Baky et al., 2020; Bakhshi et al., 2020). The virulence genes belong to various pathotypes of *E. coli*, but most are related to extra-intestinal pathogenic *E. coli* (ExPEC; Paniagua-Contreras et al., 2017; Najafi et al., 2019; Abd El-Baky et al., 2020). Among the *E. coli* strains, the ST69 has the most abundant and diverse virulence genes, including three toxin genes, *hlyE* (hemolysin), *astA* (heat-stable enterotoxin EAST-1), *espP* (serine protease autotransporter), suggesting the public health hazard associated with this strain. The ST69 *E. coli* is reported as the second most common cause of extraintestinal infection across the globe (Manges et al., 2019).

In some isolates, virulence genes and ARGs were co-located on the same plasmids or associated with other MGEs. For example, in ST69, *traT* (a virulence gene that helps the bacteria to survive the host serum bactericidal effect of the complement system) is located on IncFII (F2:A: B) plasmids together with *tet*(A) and *bla*_TEM-1_. Hayer et al. (2020) also reported an association between the *traT* virulence gene and IncFI plasmids from *E. coli* isolates obtained from pigs in the U.S. The virulence gene, *traT*, was indicated to be frequently associated with sepsis and urinary tract infections in humans (Nojoomi and Ghasemian, 2019; Abd El-Baky et al., 2020). Thus, co-location favors the clonal and horizontal spread of the virulence and resistance genes and may cause a significant clinical impact on people consuming raw milk. In addition to detecting the virulence gene in each strain, *in silico* assessment of the pathogenicity of the bacteria to the human host predicted that all the ESBL-*E. coli* are highly likely (probability > 0.92) to be a human pathogen (Cosentino et al., 2013). *Escherichia coli* is one of the most important milk borne human pathogens (WHO, 2018). *Escherichia coli* causes a wide range of severe infections in humans (Lim et al., 2010) and is also among the frequent cause of environmental mastitis in dairy cattle (Herry et al., 2017), along with colibacillosis in calves (Gelalcha et al., 2022). Thus, the presence of multiple ARGs, combined with the presence of virulence factors, make ESBL-*E. coli*, a greater threat to human health. Furthermore, the detection of potential pathogenic and MDR ESBL-*E. coli* isolates in this study support the argument about the possible convergence between ESBL-production and virulence (De Koster et al., 2022).

The ESBL-*E. coli* isolate that showed resistance to nine antibiotics was found to have the same resistant pattern as most *E. coli* isolates retrieved from fecal samples from the same farm (Farm A), suggesting possible fecal contamination of the bulk tank milk from the cow (unpublished data). Similarly, WGS analysis of this isolate identified 11 different ARGs, including *mph*(A; macrolide phosphotransferases), *aad*A1 (aminoglycoside nucleotidyltransferase), *dfrA* (dihydrofolate reductase), and *sul1*(dihydropteroate synthase) which was detected only in this isolate. Multiple *E. coli* isolates with the same serotype (045, H25) and sequence types (ST2325) were isolated from a fecal sample obtained from the same farm. Detection of the same serotypes and sequence of *E. coli* isolates from fecal samples strongly indicates fecal contamination of the BTM. This highlights the importance of implementing hygiene and sanitation measures to prevent contamination and reduce the risk of transmission of pathogenic or commensal MDR bacteria to humans.

Unlike ESBL-*E. coli*, the two *K. pneumoniae* isolates did not exhibit acquired multi-drug resistance phenotypes. However, the detected resistance genotypes of the *K. pneumoniae* isolates were consistent with their expressed phenotypes. Even without MDR phenotypes, the presence of ESBL-producing *K. pneumoniae* is still a concern, as it can contribute to the spread of antibiotic resistance (Navon-Venezia et al., 2017). Both *Klebsiella* strains harbored three beta-lactamase encoding genes, two ESBL genotypes, and one non-ESBL genotype. The detection of non-ESBL or narrow spectrum beta-lactamase, *bla*_TEM-1B_ in one and *bla*_SHV-2_ in the other strain is consistent with the established literature as *Klebsiella* spp. are inherently resistant to penicillin (Paterson, 2006). Detection of multiple resistance genes to beta-lactam antibiotics suggests that these isolates have accumulated multiple mechanisms for resistance to beta-lactam antibiotics. The observed ESBL phenotypes might be mediated by *bla*_CTX-M-27_ and *bla*_SHV-62_ in ST867 and *bla*_CTX-M-1_ and *bla*_SHV-40_ in the novel strain (Bush and Bradford, 2020).

Sequence typing assigned one of the *Klebsiella* isolates, BM-MK, to ST867, but its serotype was new and could not be assigned. The other isolate, DM-MK, was also not assigned to a particular sequence type or serotype, i.e., its genetic makeup or antigenic characteristics did not match any known *K. pneumoniae* subtypes based on its DNA sequence. This *K. pneumoniae* isolate was identified as a novel serotype and sequence type; it was not previously reported in the scientific literature. This finding highlights the importance of continuous monitoring and surveillance for the emergence of new strains of ESBL-producing bacteria to detect them early before their spread and inform public health efforts to control them.

In the novel strain of *K. pneumoniae*, the ESBL genotypes co-existed with resistance genotypes that mediate resistance to three CIAs, including aminoglycosides, fluoroquinolone, and macrolide. However, the corresponding resistance phenotypes were not observed against these antibiotics during AST, suggesting a lack of proper expression of the respective genes. The resistance to sulfisoxazole observed in the ST867 *Klebsiella* strain may be due to the *oqxAB* operon, a plasmid-borne multi-drug efflux pump (Li et al., 2019). The absence of known genes related to folate antagonists does not rule out the possibility of efflux pump-mediated resistance. Detection of multiple resistance genes in *K. pneumoniae* is consistent with the previous studies and reviews that identified *K. pneumoniae* as a” key trafficker” of multi-drug resistance genes (Holt et al., 2015; Navon-Venezia et al., 2017; Wyres and Holt, 2018).

The predominance of epidemic resistance plasmids (formerly known as narrow host plasmids), IncF, in these two strains of *K. pneumoniae* is consistent with the previous findings that reported a frequent association between ESBL-producing *Klebsiella* spp. and InF plasmid families (Carattoli, 2009). The IncFII(K) and IncFIB(K; both IncF plasmid families) identified in the novel strain are highly similar to (96%–99% nucleotide identity) those plasmids identified from MDR carbapenemase-producing ST258 *K. pneumoniae* isolate from a human in Italy (Garcia-Fernandez et al., 2012). This suggests closely related epidemic-resistance plasmids are circulating in both animals and humans across the globe. The col440I plasmids that carried *qnrB19* in the novel strain were similar (91% nucleotide identity) to the plasmid identified from *K. pneumoniae* isolates from human blood samples in the U.S. (Accession: CP023920.1; the data not published).

The ESBL-*K. pneumoniae* were also enriched with virulence determinants that may enhance their pathogenic potential. The siderophore receptor gene (*fyuA*), the Ferric aerobactin receptor gene (*iutA*), and the iron regulatory protein (*Irp2*) encode proteins associated with iron uptake and metabolism and thereby enhance their growth, survival and pathogenicity. Type 1 fimbriae (*FimH*) help the bacteria adhere to the mammalian host cell. The genes *ccI* (encodes for cloacin that help the bacteria establish a niche in the host environment without as much competition), *clpK1*(encodes heat shock survival AAA family ATPase that allow the bacteria adapt to elevated host body temperatures and also degrade host proteins), and *traT* (encodes outer membrane protein responsible for complement resistance) help evade the host defense mechanism and contribute to the pathogenicity (Tu et al., 2016; Sarowska et al., 2019; Juraschek et al., 2022). In addition, further analysis of the *Klebsiella* genome predicted both strains as possible human pathogens with a high probability (probability >0.87). The presence of ESBL and multiple virulence genes in *K. pneumoniae*, a frequent cause of severe infections in humans and mastitis in dairy cattle, highlights the significant public health and veterinary risk associated with these bacteria (Fuenzalida et al., 2021; Zheng et al., 2022). Monitoring and controlling the ESBL-producing Enterobacteriaceae, particularly those with increased virulence, is crucial to reduce infection risk and to improve public and animal health.

## 5. Conclusion

The results of this study revealed a high prevalence of multidrug resistant ESBL-*E. coli* and presence of ESBL-*K. pneumoniae* in bulk tank milk in East Tennessee dairy farms. These bacteria carry several resistance and virulence genes often linked to mobile genetic elements, making it easy for them to spread to other bacteria. Half of ESBL-*E. coli* isolates from this study are considered high-risk clones due to their ability to cause disease and acquire genetic traits that give them an advantage over other bacterial clones, including virulence factors, epidemic plasmids, and ARGs. Detection of these highly resistant and virulent bacteria in raw bulk tank milk threatens public health. The bacteria can cause clinical infection or transfer their resistance and virulence gene to a more clinically relevant strain. Feces were also found to be a potential source of ESBL-*E. coli,* which might be a source of milk contamination, suggesting the need for hygienic milking practices on farms. The WGS data of *E. coli* isolates revealed a diverse population of bacteria with seven distinct serotypes, six STs, and eight different cgSTs. The discovery of a new allele type for the IncFIA (HI1) plasmid in the novel ST of *K. pneumoniae* can help to further our understanding of the diversity of strains of this bacterium and its plasmids. This study indicates that raw milk is a reservoir of potentially zoonotic MDR *E. coli* and *K. pneumoniae*, carrying readily transferable resistance and virulence genes. Thus, the consumption of raw milk and milk products has a public health risk with potentially MDR infections.

## Data availability statement

The sequence data are deposited in the NCBI database under BioProject PRJNA1011909.

## Author contributions

BG: Conceptualization, Writing – original draft, Methodology, Investigation, Formal analysis, Writing – review & editing. RM: Writing – review & editing, Formal analysis. AG: Investigation, Writing – review & editing. OK: Supervision, Resources, Project administration, Funding–acquisition, Conceptualization, Writing – review & editing.

## Footnotes

^1^https://cge.food.dtu.dk/services/KmerFinder/

^2^https://cge.food.dtu.dk/services/ResFinder/

^3^https://card.mcmaster.ca/analyze/rgi

^4^https://cge.food.dtu.dk/services/PlasmidFinder/

^5^https://cge.food.dtu.dk/services/MobileElementFinder/

^6^https://cge.food.dtu.dk/services/VirulenceFinder/

^7^https://cge.food.dtu.dk/services/SerotypeFinder/

^8^https://cge.food.dtu.dk/services/MLST/

^9^https://cge.food.dtu.dk/services/cgMLSTFinder/

^10^https://cge.food.dtu.dk/services/PathogenFinder/

^11^https://cge.food.dtu.dk/services/pMLST/

^15^https://www.center4research.org/8094-2/
